# Complement receptor 3 (CR3)-dependent microglial synapse elimination drives Parkinson’s disease pathogenesis in systemic inflammation

**DOI:** 10.1038/s41419-026-08557-9

**Published:** 2026-03-25

**Authors:** Lei Cai, Yihe Zhang, Jiayi Li, Lamei Hu, Qinghao Meng, Jingyi Shao, Haotian Deng, Yiying Liu, Jiaqi Liu, Yue Liang, Nanshan Song, Jianhua Ding, Yi Fan, Ming Lu, Yinquan Fang, Gang Hu

**Affiliations:** 1 Jiangsu Key Laboratory of Neurodegeneration, Department of Pharmacology, School of Basic Medical Sciences, Nanjing Medical University, Nanjing, Jiangsu China; 2 Department of Pharmacology, Nanjing University of Chinese Medicine, Nanjing, Jiangsu China

**Keywords:** Cell death in the nervous system, Neuroimmunology

## Abstract

Although systemic inflammation has been implicated in PD pathogenesis, the underlying mechanisms remain poorly understood. In this study, we investigate the pathological events in a systemic inflammation-induced PD mouse model. We demonstrate that synaptic loss in the midbrain occurs as early as 1 day after the final lipopolysaccharide (LPS) administration, preceding dopaminergic (DA) neuron degeneration, which was observed only at later stages (14 days). Early microglial activation in the midbrain is detected, accompanied by excessive synaptic engulfment, suggesting a critical role of microglia-dependent synapse elimination in PD pathogenesis. Furthermore, we identify the complement receptor 3 (CR3) as a key mediator of microglial synaptic engulfment, revealing that its inhibition rescues synaptic integrity and prevents neurodegeneration. Our results contribute to a deeper understanding of early events of PD progression driven by systemic inflammation and provide early intervention strategies targeting microglial complement signaling to halt PD progression.

## Introduction

Parkinson’s disease (PD) is the second most common neurodegenerative disease (NDD), causing motor, non-motor, and cognitive impairments [1]. PD prevalence increases rapidly, affecting 2–3% of individuals over the age of 65 [2, 3]. The key pathological features of PD include the progressive loss of dopaminergic (DA) neurons and chronic neuroinflammation in the substantia nigra pars compacta (SNc), and abnormal aggregation of α-synuclein [1, 4]. Despite significant advances in understanding its pathology, the exact etiology and mechanisms of PD remain incompletely understood, with a complex interaction between genetic factors and environmental exposures contributing to its onset and progression [5, 6].

Growing evidence suggests the critical role of systemic inflammation in the etiology and progression of PD [7, 8]. Early-stage PD patients exhibit both central and peripheral inflammation, which correlates with neurodegeneration [7, 9]. Epidemiological studies also indicate that systemic inflammation accelerates ageing, one of the most significant risk factors for PD [10, 11]. Furthermore, bacterial infection that elevate lipopolysaccharide (LPS) levels and trigger inflammation have been associated with increased PD incidence and motor dysfunction [12–15]. Although numerous studies have confirmed the link between systemic inflammation and PD, the precise mechanisms remain poorly defined.

Microglia, the resident immune cells and professional phagocytes of the central nervous system (CNS), have long been recognized for their role in immune responses [16]. Evidence so far points to a lessened modulatory and neurotrophic function and an exacerbated pro-inflammatory state of microglia during the course of the PD [1, 17]. Recent research, however, suggests that microglia also play crucial roles in modulating neuronal activity and function [18, 19]. Excessive microglial phagocytosis of synapses has been implicated in the progression of several NDDs, such as Alzheimer’s disease (AD) and multiple sclerosis (MS) [20–24]. For example, in AD and Huntington’s disease (HD), early synapse loss is mediated by overactive microglia that excessively prune synapses [25, 26]. In PD, early synapse loss occurs in the striatal region, preceding dopaminergic neuron loss [27, 28]. Despite these findings, the role of microglia-dependent synapse elimination in systemic inflammation-induced PD remains poorly explored.

In this study, we have demonstrated that synaptic atrophy occurs early in the LPS-induced systemic inflammation models of PD, prior to DA neuron loss in the midbrain. We have also identified excessive microglia-dependent synaptic pruning as a key driver of this early synaptic damage. Furthermore, we have showed that the complement receptor 3 (CR3) mediates microglial activation, morphological changes, phagocytosis, and synaptic elimination in the early stages of systemic inflammation-induced PD, ultimately leading to DA neuron death in the later stages. Overall, our findings shed light on the pathological progression of systemic inflammation-associated PD and suggest a potential therapeutic strategy for the treatment of PD by targeting microglia-dependent synaptic elimination.

## Results

### Synapse loss precedes DA neurons degeneration in the midbrain of mice following systemic LPS application

**Fig. 1:**
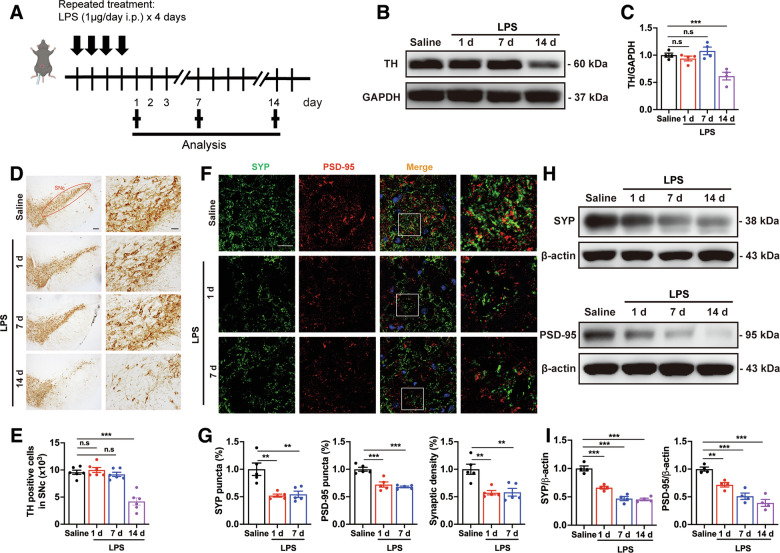
Impairment of DA neurons and synapses induced by systemic LPS challenge. **A** Schematic diagram of systemic inflammation-induced PD mouse model preferment. Biochemical analysis was conducted after the last LPS injection at 1, 7, 14 days. **B**, **C** The protein levels of TH in the midbrain of mice following systemic LPS application. *n* = 4 mice. Immunohistochemical staining (**D**) and stereological counts (**E**) of TH^+^ DA neuron in the SNc of systemic inflammation-induced PD mice. *n* = 6 mice. Scale bars, 200 μm (left panels) or 50 μm (right panels). Immunostaining (**F**) and quantification (**G**) of presynaptic (SYP) and postsynaptic (PSD-95) puncta in the SNc of LPS mice. *n* = 5 mice. Scale bar, 20 μm. **H**, **I** The expression of SYP and PSD-95 in the midbrain of LPS mice. *n* = 5 mice. Data are presented as mean ± SEM. One-way ANOVA followed by Tukey’s multiple comparisons was used for statistical analysis. ^*n.s*^*P* > 0.05, **P < 0.01, and ***P < 0.001.

Given the involvement of systemic inflammation in PD pathology, we utilized a well-established PD mouse model, in which mice were administered intraperitoneal LPS for four consecutive days [29]. Mice were sacrificed at 1, 7, and 14 days post-LPS administration (LPS-1d, LPS-7d, LPS-14d) to evaluate DA neuron integrity using immunoblotting and immunohistochemistry staining of tyrosine hydroxylase (TH) (Fig. 1A). TH protein expression in the midbrain and the number of DA neurons in the SNc of mice was significantly reduced at day 14 post-LPS, compared to control mice (Fig. 1B–E). However, no significant changes in TH-positive DA neurons were observed at day 1 and 7 in LPS mice (Fig. 1B–E). These data demonstrate that DA neuron degeneration occurs at later stages of systemic LPS-induced inflammation, while no major loss is observed during the early and middle stages.

Synaptic dysfunction often precedes neuronal death in NDDs [30]. Thus, we used double immunofluorescence staining to quantify the synapse density by using synaptophysin (SYP) as a presynaptic marker and postsynaptic density-95 (PSD-95) as a postsynaptic marker. Both SYP and PSD-95 densities, as well as their colocalization were significantly decreased in the SNc of mice after the last LPS administration for 1 and 7d, compared to controls (Fig. 1F, G). Immunoblot results also showed that the protein levels of SYP and PSD-95 in the midbrain of LPS mice were lower than that in the control mice at all time points studied (Fig. 1H, I). These results demonstrate that synaptic loss in the midbrain precedes DA neuron degeneration in systemic LPS-induced PD models.

### Systemic LPS application induces microglial activation and microglia-mediated synapse phagocytosis in the early-stages

**Fig. 2:**
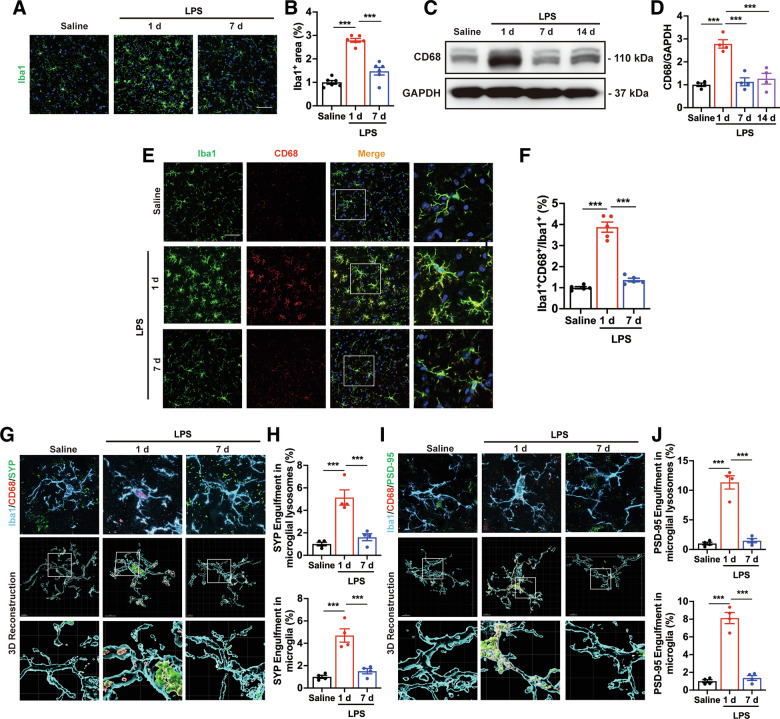
Microglial activation and phagocytosis of synapses in systemic LPS-induced PD mouse models. Immunostaining (**A**) and quantitative fluorescence analysis (**B**) of Iba1^+^ microglia in the SNc of LPS-treated mice at 1 and 7 days. *n* = 6 mice. Scale bar, 50 μm. **C**, **D** The protein levels of CD68 in the midbrain of mice after 1,7 and 14 days of systemic LPS challenge. *n* = 4 mice. **E**, **F** CD68 expression on iba1^+^ microglia in the SNc of LPS-treated mice at 1 and 7 days. *n* = 5 mice. Scale bar, 50 μm. **G**, **I** 3D reconstruction of individual microglia (Iba1^+^) engulfing presynaptic (SYP^+^, **G**) and postsynaptic (PSD-95^+^, **I**) puncta, along with phagolysosomal structures (CD68^+^), in the SNc of systemic inflammation-induced PD mice. Scale bar, 5 μm. **H** Quantitative data of SYP^+^ puncta within microglial lysosomes (*n* = 139 cells from 12 mice, 4 mice per group) and SYP+ puncta within microglia (*n* = 139 cells from 12 mice, 4 mice per group) per cell shown in (**G**). **J** Quantitative data of PSD-95^+^ puncta within microglial lysosomes (*n* = 144 cells from 12 mice, 4 mice per group) and PSD-95^+^ puncta within microglia (*n* = 144 cells from 12 mice, 4 mice per group) per cell shown in (**I**). Data are presented as mean ± SEM. One-way ANOVA followed by Tukey’s multiple comparisons was used for statistical analysis. ***P < 0.001.

Microglia sensitive to changes in the brain parenchyma induced by various stimuli, including systemic inflammation [7]. We determined the activation of microglia using immunofluorescence staining of ionized calcium-binding adapter molecule 1 (Iba1). As expected, systemic LPS treatment triggered significant microglial activation in the SNc of mice at LPS-1d and LPS-7d, with activation levels peaking at LPS-1d (Fig. 2A, B). As microglial phagocytosis plays a crucial role in central nervous system (CNS) disorders [31], we determined microglial phagocytic activity by measuring Cluster of Differentiation 68 (CD68) protein levels, a marker for macrophage/microglial lysosomes. CD68 expression was significantly elevated in the midbrain of LPS mice at day 1, but recovered to baseline at days 7 and 14 (Fig. 2C, D). Immunofluorescence staining also showed that CD68 levels were significantly increased in microglia of the SNc after 1 day of LPS treatment, but not after 7 days (Fig. 2E, F). These finding suggest systemic LPS leads to the activation and the phagocytotic abilities of microglia in the midbrain at the early stages, but this effect wanes over time.

Recently, research has indicated that microglia play an important role in synapse elimination through excessive synaptic pruning [18, 32]. To investigate microglia-mediated synapse phagocytosis, we then co-labeled Iba1 and CD68 with SYP and PSD-95 and analyzed their localization within microglial lysosomes. Three-dimensional (3D) reconstructions showed an increased engulfment of synaptic components by microglia in the SNc of LPS mice at 1 day post-treatment (Fig. 2G–J). Additionally, the expression of lysosome-associated genes, including Rab5b and Rab7, was significantly upregulated in the midbrain of LPS-treated mice at day 1 (Supplementary Fig. 1). However, by LPS-7d, the engulfment of synaptic structure within microglial lysosomes and the levels of lysosome-related gene had returned to control levels (Fig. 2G–J; Supplementary Fig. 1). These results indicate that excessive microglial phagocytosis drives early synapse loss in systemic LPS-induced PD.

### Systemic LPS challenge activates complement pathway in the midbrain

**Fig. 3:**
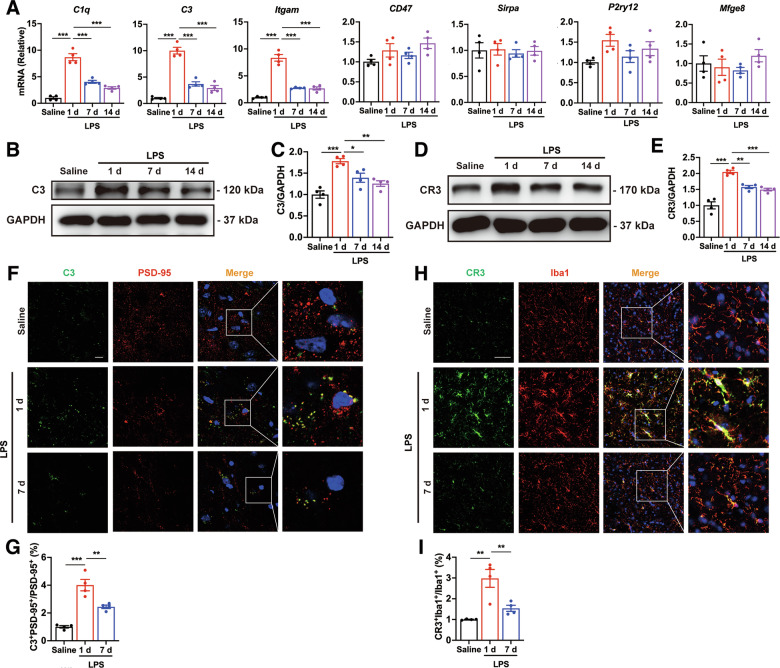
Activation of complement pathway induced by systemic LPS application. **A** The mRNA expression of complement cascade signals and phagocytotic receptors in the midbrain of systemic inflammation-induced PD mice. *n* = 4 mice. The protein levels of C3 (**B**, **C**) and CR3 (**D**, **E**) in the midbrain of LPS mice. *n* = 4 mice. Immunostaining (**F**) and quantification (**G**) of colocalized C3 and PSD-95 puncta in the SNc of LPS mice after stimulation for 1 and 7 days. *n* = 4 mice. Scale bar, 10 μm. **H**, **I** CR3 expression in microglia in the SNc of LPS-treated mice at 1 and 7 days. *n* = 4 mice. Scale bar, 50 μm. Data are presented as mean ± SEM. One-way ANOVA followed by Tukey’s multiple comparisons was used for statistical analysis. *P < 0.05, **P < 0.01, and ***P < 0.001.

We next sought to explore the underlying mechanisms of microglia-dependent elimination of synapses in the systemic inflammation induced PD models. As the complement cascade signals and phagocytotic receptors play crucial roles in microglia-mediated synapses elimination [32], we firstly measured the mRNA expression of complement components (C1q, C3, CR3), cluster of differentiation 47 (CD47), signal regulatory protein α (Sirpa), purinergic receptor P2Y12 (P2ry12r) and milk fat globule EGF factor 8 (Mfge8). Among these genes, the transcript levels of C1q, C3 and CR3 (encoded by *Itgam*) were markedly elevated in the midbrain of LPS-treated mice, with peak expression at LPS-1d (Fig. 3A). Within the complement cascade, C3 plays a central role in initiating downstream signaling and localizes on synapses, mediating microglia-dependent engulfment of synapses through CR3 receptors, which are exclusively expressed on microglia [25, 29, 33]. We also found that, compared with the control group, the protein levels of both C3 and CR3 were significantly increased in the midbrain of mice after LPS stimulation, with peak expression at day 1, followed by a slight reduction at days 7 and 14 (Fig. 3B–E). Furthermore, elevated C3 levels were localized to PSD-95-positive synapses (Fig. 3F, G), while increased CR3 expression was enriched on Iba1-positive microglia at LPS-1d (Fig. 3H, I). These data demonstrate that systemic inflammation activates the C3/CR3 complement pathway, which correlates with microglial-mediated synapse elimination in the early stages of systemic LPS-induced PD.

To investigate other mechanisms contribute to the reduction of DA neurons related to complement pathway at day 14 after consecutive doses of LPS, the levels of pro-inflammatory markers, purinergic receptors, and extracellular matrix components were performed using transcriptome analysis. Neuroinflammation, characterized by robust production of pro-inflammatory factors, fuels neurodegeneration in PD [34, 35]. All pro-inflammatory markers tested, including *Csf1*, *Il1b*, *Il6*, and *Tnfa* genes were markedly elevated in the midbrain of LPS-treated mice, with peak expression at day 1, followed by a reduction at days 7 and 14 (Supplementary Fig. 2A). This data aligns well with the activation of the complement pathway, suggesting that the complement pathway may also contribute to the production of inflammatory cytokines in the early- and intermediate- stages of systemic LPS-induced PD.

Recent work has identified that purinergic receptors are critically involved in microglial activation, neuroinflammatory responses and neurodegeneration [36, 37]. The formation of perineuronal nets (PNNs), a unique extracellular matrix components (ECMs) subtype is associated with synaptic dysfunction and neuron damage in neurological diseases [38]. However, no significant differences were found in the expression of purinergic receptors (*Adora2a*, *P2ry2*, *P2rx4* and *P2rx7*) and PNNs components (*Aggrecan*, *Brevincan* and *Neurocan*) studied between the control and LPS mice at all time points tested (Supplementary Fig. 2B, C). Furthermore, we have found that the mRNA levels of Matrix Metalloproteinase (MMP)-9, a primary degradative enzyme for PNNs, is elevated during the early phase of systemic LPS-induced PD models, while it returns to baseline levels in the intermediate and late stages (Supplementary Fig. 2C). These data suggest that systemic inflammation may not affect the formation of PNNs but could promote their degradation.

### C3/CR3 pathway mediates synaptic elimination by microglia in vitro

**Fig. 4:**
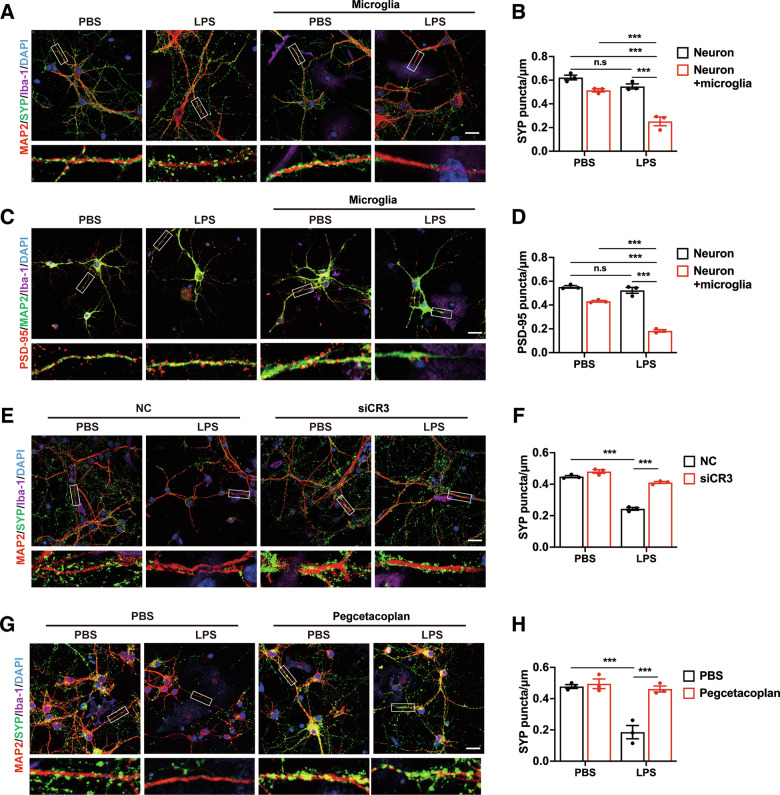
Effects of C3/CR3 pathway on microglia-dependent synapses elimination in microglia-neuron coculture system. Immunostaining (**A**, **C**) and quantification (**B**, **D**) of presynaptic (**A**, **B**) and postsynaptic (**C**, **D**) puncta in VM neurons and microglia-neuron coculture, with or without LPS treatment for 24 h. Scale bar, 20 μm. *n* = 3 independent experiments. Immunostaining (**E**) and quantification (**F**) of SYP puncta in VM neurons coculture with CR3-deficiency microglia. Immunostaining (**G**) and quantification (**H**) of SYP puncta in microglia-neuron coculture, with or without C3 inhibitor pretreatment. Scale bar, 20 μm. *n* = 3 independent experiments. Data are presented as mean ± SEM. Two-way repeated-measures ANOVA followed by Tukey’s multiple comparisons were used for statistical analysis. ^n.s^*P* > 0.05, and ***P < 0.001.

To explore the relationships between C3/CR3 pathway, synaptic loss, and microglia, we constructed a microglia-neuron coculture system, where ventral mesencephalic (VM) neurons were cocultured with microglia at a 3:1 ratio, with or without LPS stimulation. Directly LPS stimulation of primary cultured neuron failed to affect the density of synapses (Fig. 4A–D). In the microglia-neuron coculture system, LPS challenge significantly reduced the synaptic density of VM neuron, as evidenced by decreased SYP and PSD95 puncta (Fig. 4A–D).

We then determinate whether C3/CR3 pathway was involved in LPS-induced the synaptic impairment by microglia. The transcript levels of C3 and CR3 were significantly enhanced in microglia after LPS stimulation (Supplementary Fig. 3). CR3 deficiency by siRNA (Supplementary Fig. 4) significantly reduced the mRNA levels of C1q and C3 (Supplementary Fig. 5A, B), indicating that CR3 is required for the full activation of the complement cascade. CR3 knockdown and C3 inhibitory peptide pegcetacoplan acetate clearly normalized the reduction of synapses in the microglia-neuron coculture system after LPS stimulation (Fig. 4E–H). The neurons exhibited apoptotic features and impaired MAP2^+^ neurite length in the microglia-neuron coculture system upon LPS stimulation, which were inhibited by the CR3 knockout in microglia and C3 inhibitor pretreatment (Supplementary Fig. 6). These results indicate that C3/CR3 pathway is responsible for microglia-mediated synaptic elimination and neuron damage induced by LPS.

### Microglial CR3 knockdown mitigates the impairment of synapses and DA neurons induced by systemic LPS challenges

**Fig. 5:**
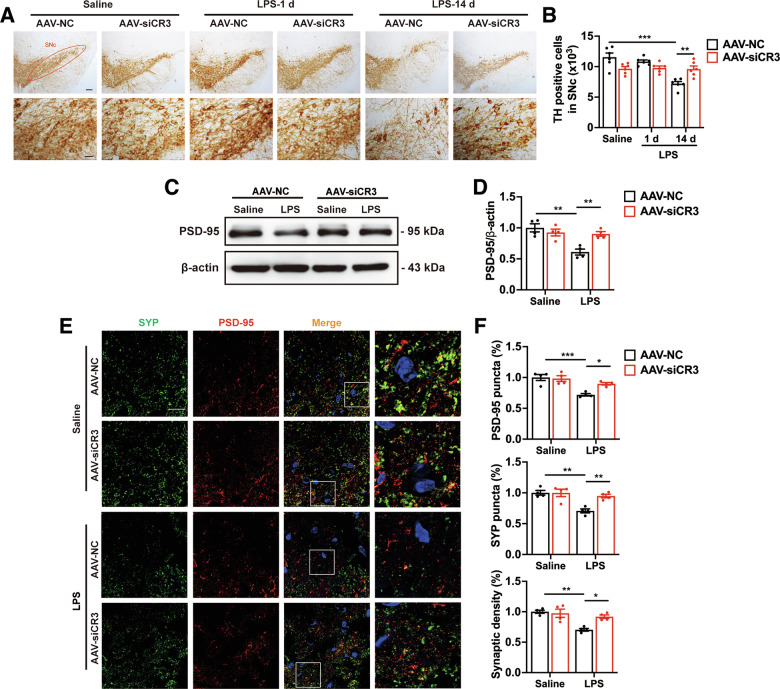
Effects of CR3 depletion in microglia on DA neurons and synapses loss in systemic LPS-induced PD mouse models. Immunohistochemistry (**A**) and stereological quantification (**B**) of TH^+^ DA neuron in the SNc of systemic LPS-induced PD mice. *n* = 6 mice. Scale bars, 200 μm (upper panels) or 50 μm (lower panels). **C**, **D** The protein levels of PSD-95 in the midbrain of LPS-treated mice. *n* = 4 mice. Immunostaining (**E**) and quantification (**F**) of presynaptic and postsynaptic puncta in the SNc of mice after systemic LPS challenges. Scale bar, 20 μm. *n* = 4 mice. Data are presented as mean ± SEM. Two-way repeated-measures ANOVA followed by Tukey’s multiple comparisons was used for statistical analysis. *P < 0.05, **P < 0.01, and ***P < 0.001.

To further elucidate the role of CR3 pathway in vivo (Supplementary Fig. 7A), we next used adeno-associated viruses (AAVs) carrying the microglia-specific promoter Iba1 to deliver siRNA (Supplementary Fig. 7B) to knock down CR3 in microglia as confirmed by immunostaining (Supplementary Fig. 7C–H). Consistent with the data in vitro, AAV-mediated CR3 deficiency in microglia significantly decreased the transcript levels of C1q and C3 in the midbrain of mice after systemic LPS challenge (Supplementary Fig. 5C, D). As expected, systemic LPS challenge caused remarkable DA neuron loss in the SNc of mice at day 14, but not at day 1, as assessed by immunohistochemistry for TH (Fig. 5A, B). Notably, CR3 knockdown in microglia significantly relieved DA neuron death in the SNc at LPS-14d (Fig. 5A, B). Furthermore, AAV-mediated CR3 deficiency in microglia led to a marked increase in PSD-95 protein levels in the midbrain of LPS mice at day 1 (Fig. 5C, D), as well as an enhancement in the density of SYP and PSD-95, and their colocalization in the SNc (Fig. 5E, F). These findings suggest that microglial CR3 pathway play a critical role in systemic LPS administration induced DA neuron loss and synaptic impairment.

### The CR3 pathway activates microglia and induces synapses elimination in systemic LPS-induced PD models

**Fig. 6:**
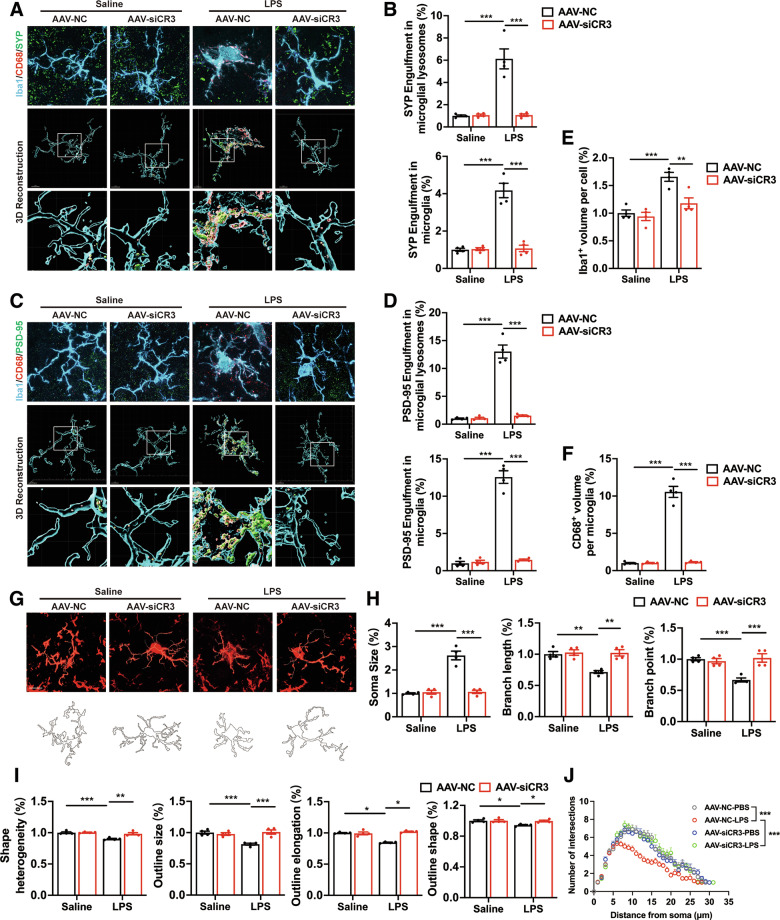
Effects of microglial CR3 knockdown on microglia-mediated engulfment of synapses in systemic LPS-induced PD mouse models. 3D reconstruction of individual microglia (Iba1^+^) engulfing presynaptic (SYP^+^, **A**) and postsynaptic (PSD-95^+^, **C**) puncta, with phagolysosomal structures in the SNc of LPS-treated PD mice. Scale bar, 5 μm. **B** Quantitative data of SYP^+^ puncta within microglial lysosomes (*n* = 217 cells from 16 mice, 4 mice per group) and SYP^+^ puncta within microglia (*n* = 215 cells from 16 mice, 4 mice per group) per cell shown in (**A**). **D** Quantitative data of PSD-95^+^ puncta within microglial lysosomes per cell (*n* = 178 cells from 16 mice, 4 mice per group) and PSD-95^+^ puncta within microglia (*n* = 173 cells from 16 mice, 4 mice per group) shown in (**C**). Volume of Iba-positive microglia (**E**) (*n* = 273 cells from 16 mice, 4 mice per group) and CD68-positive phagolysosomal structures masked in microglia (**F**) (*n* = 309 cells from 16 mice, 4 mice per group) in the SNc of mice after systemic LPS challenges. **G** Representative images of Iba1 immunostaining of microglia in the SNc of saline- and LPS-treated mice after AAV injection. Scale bar, 10 μm. **H** Quantification of soma size (*n* = 235 cells from 16 mice, 4 mice per group), total branch length (*n* = 223 cells from 16 mice, 4 mice per group), and branch points (*n* = 199 cells from 16 mice, 4 mice per group) of Iba1^+^ cell. **I** Fractal analysis of lacunarity (*n* = 260 cells from 16 mice, 4 mice per group), density (*n* = 259 cells from 16 mice, 4 mice per group), span ratio (*n* = 276 cells from 16 mice, 4 mice per group), and circularity (*n* = 250 cells from 16 mice, 4 mice per group) of Iba1^+^ cell. **J** Sholl analysis of Iba1^+^ microglia in the SNc of LPS-treated mice. *n* = 119 cells from 16 mice, 4 mice per group. Data are presented as mean ± SEM. Two-way repeated-measures ANOVA followed by Tukey’s multiple comparisons was used for statistical analysis. *P < 0.05, **P < 0.01, and ***P < 0.001.

We next examined the effects of CR3 pathway on microglia-mediated synapse elimination within the context of systemic LPS-induced PD models. AAV-mediated CR3 deficiency in microglia significantly reduced the engulfment of the presynaptic protein SYP and postsynaptic protein PSD-95 in CD68-positive lysosomes of microglia in the SNc of LPS stimulated PD mice at day 1 (Fig. 6A–D). In addition, the volumes of Iba1-positive microglia and CD68-positive lysosomes in microglia were also significantly reduced by microglial CR3 knockdown in the SNc of LPS mice (Fig. 6E, F). These findings confirm that CR3 is essential for microglia-mediated synapse phagocytosis during systemic inflammation.

Since microglial morphology is closely correlated with their activation state [39], we analyzed the morphological changes in microglia. Under LPS stimulation, microglia exhibited increased soma volume, reduced branching complexity, and shorter processes, indicating a shift to a hyperactivated state (Fig. 6G, H). Fractal analysis further revealed a reduction in lacunarity, density, span ratio, and circularity of microglia in the SNc of LPS-treated mice, which reflects cell shape heterogeneity and the size, elongation and shape of cell outline (Fig. 6G, I). Moreover, Sholl analysis revealed that LPS treatment led to a less ramified morphology in microglia, further supporting their activated state (Fig. 6J). Importantly, all of these changes in microglial morphology induced by LPS stimulation were clearly reversed by microglial CR3 knockdown mediated by AAVs (Fig. 6G–J). Together, these results suggest that the CR3 is a key regulator of microglial activation and excessive synapse elimination in systemic LPS-induced PD models.

## Discussion

**Fig. 7:**
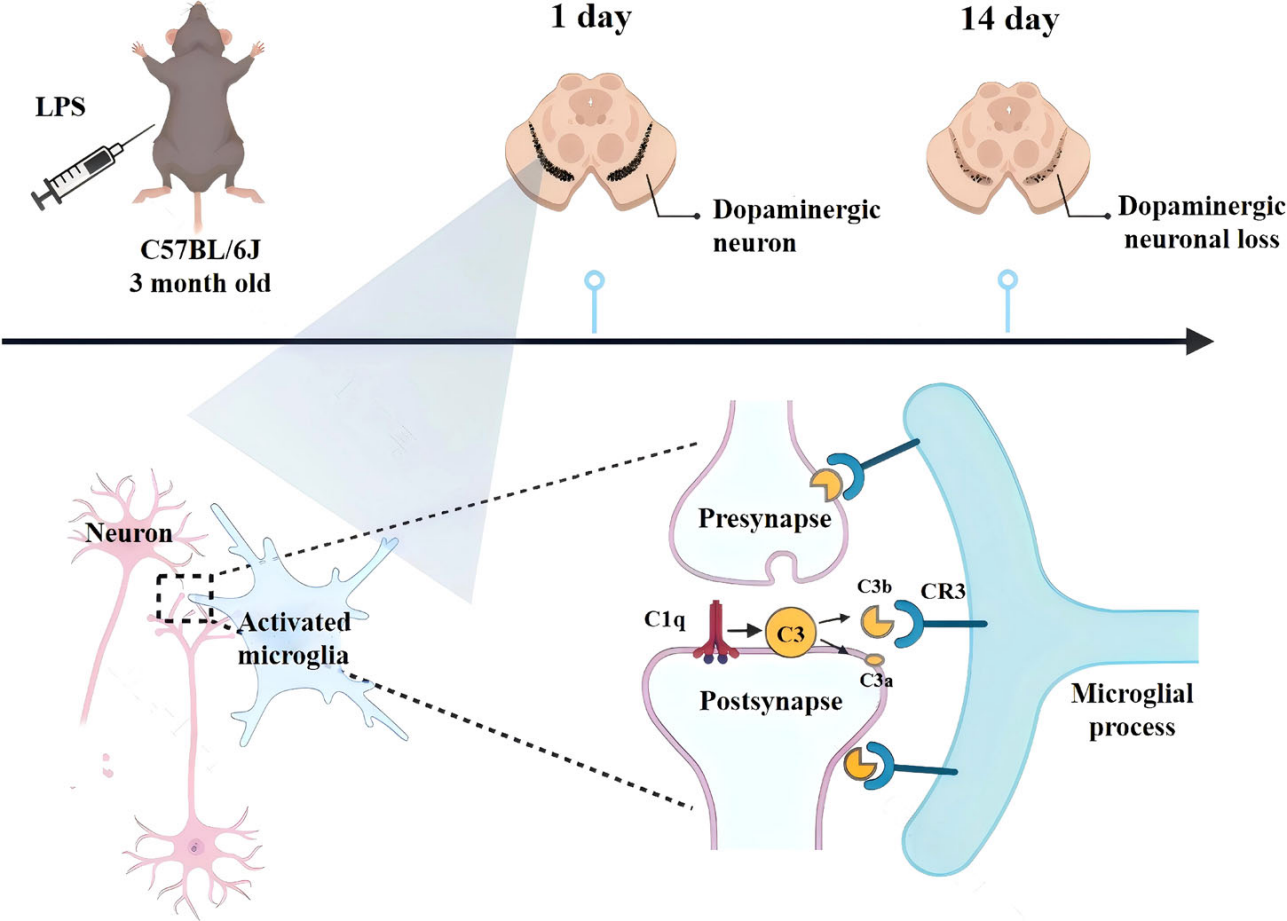
A schematic diagram showing pathological process and the roles of CR3 pathway in systemic LPS-induced PD mouse models. Microglia were activated and eliminated synapses in the early stage mediated by CR3 pathway, and then induced the loss of DA neurons in the late stage of systemic inflammation-induced PD mouse models.

This study explores the pathophysiological mechanisms underlying PD associated with systemic inflammation, emphasizing the interplay between synaptic dysfunction, microglial activation, and DA neuron degeneration (Fig. 7).

As an initial approach to explore the pathological development of PD associated with systemic inflammation, we compared the loss of DA neurons and the synaptic dysfunction in an LPS-induced PD model. Both human and experimental studies have highlighted the relevance of LPS models in understanding the characteristics and pathogenesis of clinical PD [40]. We demonstrate that the synaptic loss occurs as early as 1 day after the last application of LPS, whereas the death of DA neurons in the midbrain is observed at later stages (14 days). These data indicate that the synaptic dysfunction is an early pathological feature in systemic inflammation-induced PD mice, occurring prior to DA neuron death. Importantly, this suggests that therapeutic strategies targeting synaptic integrity in the early stages of PD may mitigate downstream neuronal degeneration and disease progression.

We have also identified the critical role of microglia in mediating synapse elimination during the early stages of PD induced by systemic inflammation. Previous studies have demonstrated that microglia are the key cell types associated with the pathogenesis of PD by modulating CNS inflammation [1, 41–43]. In the present study, we have observed that microglia were activated and endocytosed synapses in the midbrain of LPS-treated PD mice on day 1, with this activity subsiding by day 7. These findings suggest a transient but excessive microglial response to systemic inflammation, resulting in early synaptic damage and subsequent DA neuron loss. Although previous research has demonstrated that microglial synaptic pruning contributes to neurodevelopment, aging, and various CNS disorders [44–46], its dysregulation in PD has remained underexplored. Beyond systemic inflammation-induced PD, microglial activation has also been observed from the earliest stages of sporadic PD [1, 17, 47]. However, there is a scarcity of studies performed investigating the role of microglia in the early stages of PD. Thus, our data suggest that, in addition to mediating CNS inflammation, microglia-dependent synapse elimination plays a key role in the early pathology of PD.

Our data have revealed that CR3 pathway as a key driver of microglial-mediated synapse elimination, leading to subsequent DA neurons death in the systemic inflammation-induced PD models. Transcriptional profiling and immunostaining data indicate that complement C3 in synapse and CR3 in microglia were upregulated in the midbrain of LPS mice at early stages. Furthermore, microglia-specific CR3 ablation significantly alleviated microglial activation and morphologic change, microglia-dependent synapse loss and DA neuron degeneration in systemic inflammation-induced PD mouse models, both in vivo and in vitro. These findings align with the microglial complement-phagosome pathway, in which CR3 serves as the key phagocytic receptor for C3-opsonized synapses. While C3 knockout is known to be protective [29], our study significantly extends this by demonstrating that microglia-specific CR3 knockdown and pharmacological C3 inhibition are sufficient to prevent neurodegeneration. Notably, CR3 deficiency also reduced the transcript levels of C3 and C1q, suggesting that CR3 signaling is required for the full activation of the complement cascade, potentially forming a positive feedback loop. The complement system is widely recognized for its ability to mediate innate immune responses to invading microbes and adaptive immune responses to infection or injury [48]. Recent studies also suggest that C3/C3R signaling regulates neurodevelopment and neuroplasticity in the adult and aging brain through synaptic pruning. For instance, when synaptic activity is decreased, microglia engulf synapses in a CR3- and C3-dependent manner in the postnatal retinogeniculate system [49]. In the context of systemic inflammation, peripheral immune challenges can amplify complement pathway activation within the CNS and promote microglia-synapse contacts and neuronal damage [50–52]. Dysregulation in complement-mediated synaptic pruning has been implicated in several CNS disorders such as depression, epilepsy, schizophrenia, MS, AD and HD [22, 25, 26, 50, 51, 53, 54]. Notably, although other mechanisms such as MMP-9-mediated degradation of PNNs were also modulated by inflammation, their temporal patterns differed from the persistent synapse loss, further underscoring the central role of CR3 pathway in synaptic elimination. Our findings extend prior work by demonstrating that complement-mediated synapse elimination drives early synaptic dysfunction in PD and suggests a therapeutic intervention by targeting CR3 pathway at early stages.

In summary, this study reveals that microglia-dependent synapse elimination serves as a key and early pathological mechanism of synapse loss that occur in PD models induced by systemic inflammation, occurring before DA neuron death. Furthermore, it establishes that microglial CR3 pathway plays an essential role in the induction of microglia-dependent synapse elimination in the early stages of PD. This study advances our understanding of PD pathogenesis and provides a foundation for developing novel therapeutic strategies aimed at preserving synaptic and neuronal integrity during the early stages of the disease.

## Materials and methods

### Antibodies and reagents

All reagents, commercial kits, and antibodies are listed in Supplementary Table. 1.

### Animals

C57BL/6J mice (20 ± 2 g) used in this study were obtained from the Experimental Animal Center of Nanjing Medical University (Nanjing, China). Mice were housed under standard conditions (temperature: 25 °C ± 2 °C; light/dark cycle: 12/12 h; ad libitum feeding). All animal studies were conducted in accordance with ethical guidelines and were approved by Institutional Animal Care and Use Committee of the Nanjing Medical University Experimental Animal Department (IACUC-2205069).

### Systemic inflammation-induced PD models

To induce DA neurodegeneration in the midbrain, we used a repeated systemic LPS treatment, as previously described [29]. Briefly, mice (male, 3 months old) were randomly divided into groups (*n* = 10) and administered with LPS (1 mg/kg, i.p., Sigma, USA) once daily for 4 consecutive days. Mice were sacrificed on days 1, 7, and 14 after the last LPS injection, and brain tissues were collected for subsequent analyses, including immunoblotting, reverse transcription quantitative polymerase chain reaction (RT-qPCR), immunohistochemistry (IHC) and immunofluorescence (IF).

### Injection of AAVs

The AAV9 viruses (HANBIO, China) expressing mouse CR3 siRNA (AAV-CR3, 1*10^12) or control siRNA (AAV-NC, 1*10^12) (Supplementary Table. 2) under the Iba1 promoter were microinjected bilaterally into C57BL/6J mice (male, 3 months old, *n* = 10 in each group) at the SNc (AP: −3.0 mm; ML: ±1.3 mm; DV: −4.2 mm) at 0.2 μL/min for 5 min (total 1 μL AAVs) using a stereotaxic apparatus. For visualization, AAV-ZsGreen (hereafter referred to as GFP for simplicity) was co-administered. After 4 weeks, the mice were administered with LPS to develop systemic inflammation-induced PD models. The mice were randomly assigned to each group.

### Brain tissue processing

Mice were transcardially perfused with cold phosphate-buffered saline (PBS) or PBS plus 4% paraformaldehyde (PFA) as previously described [55]. For IHC and IF, the brains were harvested, post-fixed with 4% PFA overnight at 4 °C, and dehydrated in 20 and 30% sucrose for 1 week. Frozen 20 or 30 μm brain sections were sliced by a Leica CM1860 cryostat (German) and stored at −80 °C until use. For immunoblotting and RT-qPCR, the brains were harvested, and the midbrain were dissected and stored in Eppendorf tubes at −80 °C.

### Immunohistochemistry

Midbrain sections deparaffinized with 3% H_2_O_2_ for 30 minutes, blocked by 5% bovine serum albumin (BSA) in PBS containing 0.03% Triton X-100 for 60 min, and then incubated with mouse anti-tyrosine hydroxylase (TH) antibody (1:500, Santa Cruz Biotechnology, sc-25269, USA) at 4 °C overnight as described [56]. After washing, the sections were incubated with HRP-conjugated goat anti-mouse antibody (1:1000, proteintech, SA00001-1, China) for 60 min, followed by DAB staining (MXB Biotechnologies, DAB-2031, China). Images were captured using a stereomicroscope (Olympus, Japan), and TH-positive cells in the substantia nigra pars compacta (SNc) were quantified stereologically using MBF Bioscience Stereo Investigator software (MBF Bioscience, USA). Investigators were blinded to the group allocation during analysis.

### Cell cultures and treatments

Primary neuron and microglia cultures were conducted as described in previous studies [57, 58]. For primary microglia cultures, the meninges and vessels were removed from neonatal mouse brains (postnatal days 0-3), and tissues were treated with 0.25% trypsin/EDTA (Solarbio, 9002-07-7, China). Cells were suspended in complete medium [Dulbecco’s modified Eagle medium (DMEM)/F12 medium (Gibco, 12500-062 USA), 10% fetal bovine serum (FBS, Gibco, 16000-044) and 1% streptomycin-penicillin mixture (NCM Biotech, China) and cultured in T75 flasks pre-coated with cell adherent reagent (C1010, Applygen, China). The medium was changed every 3 days for 10–14 days.

For primary cultured neurons, fetal mouse brains (embryonic days 15-16) were collected, and the ventral midbrain (VM) was dissected and treated with 0.125% trypsin/EDTA, and then the cells were plated on poly-L-lysine (PLL, 0.05 mg/mL, P7405, Sigma)-coated glass slides in 24-well plates in DMEM/F12 supplemented with 10% FBS and 1% streptomycin/penicillin for 6 h at 37 °C in 5% CO_2_. Then, the media were replaced with neurobasal media (Gibco, 21103-049) supplemented with 2% B27 (Gibco, 17504044) and 0.5 mM glutamine (Gibco, 25030081). The medium was half-changed every 3 days for 7 days.

Microglia and VM neuron cocultures were established as described [59]. When microglia reached maturity, the flasks were shaken gently to collect the microglia. After centrifugation at 500 × *g* for 10 min, the cells were added to primary neurons at a 1:3 microglia-to-neuron ratio for 3 days with or without LPS (100 ng/mL, Sigma) stimulation. Pegcetacoplan acetate (1 μM, MedChemExpress, HY-P3252A, USA) were used to pretreat microglia-neuron coculture system for 1 hour before LPS stimulation according to the manufacturer’s instructions. Synaptic density was visualized by immunofluorescence staining.

### Cell transfection

Primary microglia were transfected with siRNAs targeting CR3 (GenePharma) (Supplementary Table. 2) using Lipofectamine RNAiMAX (Invitrogen, Life Technologies, 13778-150, USA) in Opti-MEM medium (Gibco, 31985-070) according to the manufacturer’s instructions. After 8 h, the transfection mixture was replaced with complete medium, and cells were cultured for an additional 40 h before co-culturing with neurons.

### Immunofluorescence staining and analysis

Immunofluorescence staining was performed as previously described [60]. Frozen 20-μm-thick brain sections were blocked with 5% BSA (Yi Fei Xue Bio Technology, TV0815) or goat serum (Boster Biological Technology, 18F29C09) containing 0.03% Triton X-100 for 60 min and then incubated with primary antibodies at 4 °C overnight. The following primary antibodies were used: rabbit anti-Iba1 antibody (1:500), mouse anti-CD68 antibody (1:50), mouse anti-PSD-95 antibody (1:100), rabbit anti-Synaptophysin antibody (1:200), rabbit anti-C3 antibody (1:200), rabbit anti-CR3 antibody (1:200). After washing, sections were incubated the secondary antibodies for 60 min as follows: Alexa Fluor 555 goat anti-mouse antibody (1:1000) and Alexa Fluor 488 goat anti-rabbit antibody (1:1000). Nucleus were stained with Hoechst (1:1000 diluted in PBS, Millipore, USA) for 10 min. Fluorescence images were acquired using a confocal laser-scanning microscope (Zeiss LSM700, Oberkochen, Germany) and analyzed by ImageJ software.

For tyramide signal amplification (TSA) coupled with multiplex fluorescence staining [61], mix cultured cells were fixed with 4% PFA for 30 min. Brain slices and fixed cells were treated with 0.03% H_2_O_2_ for 15 min to quench endogenous peroxidase activity. After blocking with goat serum containing 0.03% Triton X-100 for 60 minutes, slices were then incubated overnight at 4 °C with mouse anti-CD68 antibody or rabbit anti-MAP2 antibody (1:500). After washing, slices were then treated with a horseradish peroxidase conjugated anti-mouse antibody (1:1000) for 1 h, and then incubated in a biosignal tyramide-fluorescein (BT-FITC) conjugate (1:100) for 20 s. The reaction was stopped by immersing the slices in sterilized distilled water, after which they were coverslipped with a mounting medium containing 2.5% 1,4-diazabicyclo[2.2.2]octane (DABCO; Millipore) in glycerol (Millipore) for 1 h. Following the first incubation, slices were rinsed and then treated overnight at 4 °C with mouse anti-PSD-95 antibody or rabbit anti-Synaptophysin antibody. After washing, sections were incubated overnight at 4 °C with rabbit anti-Iba1 antibody, followed by Alexa Fluor 647-conjugated goat anti-rabbit antibody for 1 h. Hoechst staining was then performed for 10 min to label cell nuclei.

Fluorescence images were captured by a confocal laser-scanning microscope (Zeiss LSM800, Oberkochen, Germany). To quantify synaptic terminals, synapse co-localization was determined by analyzing the co-localization of the presynaptic marker synaptophysin and the postsynaptic marker PSD-95 in the midbrain of mice using ImageJ software. To assess the engulfment of synaptic terminals by microglia, 3D reconstructions of Iba1^+^ microglia were generated using the “Surface” function in Imaris software. The Iba1^+^ microglia surface was used to mask the CD68 channel. The 3D reconstructions of the masked CD68 channel were then used to further mask the PSD-95 or synaptophysin channel. The “Surface” function was applied to count the volume of synaptic puncta entirely within the Iba1^+^CD68^+^ surface [49, 62]. All fluorescence image analyses were performed in a blinded manner to the experimental groups.

### Microglia morphology analysis

Brain sections were sained with Iba1, and z-stack images were obtained by a confocal laser-scanning microscope (Zeiss LSM800) and analyzed using ImageJ software. Branch length and endpoint were quantified using the “Skeleton Analysis” function. Lacunarity, density, span ratio, and circularity were measured using the “Fractal Analysis” function [63]. Sholl analyses were conducted by measuring intersections at 1 µm intervals starting from soma using “Sholl Analysis” function [64]. Investigators were blinded to the group allocations.

### Immunoblotting

Tissues or primary cultured cells were lysed in RIPA buffer supplemented with protease inhibitor cocktail (Invitrogen, A32965, USA) and then centrifuged at 16,000 × *g* for 15 min at 4 °C. The protein concentrations in the supernatants were determined by BCA Protein Assay (KeyGen BioTECH, KPG903, China). Equal amounts of protein (30 μg) were separated by SDS/PAGE on an 8–12% gradient separating gels and transferred onto polyvinylidene difluoride (PVDF) membrane (PALL Gelman, USA) using Mini-PROTEAN® Tetra Handcast Systems and Mini Trans-Blot® Cell (Bio-Rad Laboratories, USA). Membranes were blocked for 1 h with 5% fat free milk and incubated with primary antibodies overnight at 4 °C. Antibodies used included: mouse anti-TH antibody (1:1000), mouse anti-CD68 antibody (1:500), mouse anti-PSD-95 antibody (1:500), rabbit anti–Synaptophysin antibody (1:30000), rabbit anti-C3 (1:1000), rabbit anti-CR3 (1:1000), mouse anti-β-actin (1:3000) and mouse anti-GAPDH (1:3000). After washing, the membranes were incubated with HRP-conjugated secondary antibody (1:5000) for 1 h. Bands were detected by enhanced chemiluminescence western blot detection reagents (Pierce) and analyzed with the ImageQuant™ LAS 4000 imaging system (GE Healthcare, USA) and Bio-Rad Gel Doc XR documentation system.

### RT-qPCR

Total RNA was extracted from mouse brain tissues or cultured microglia using Trizol reagent (Life Ambion, 15596018,China), and reverse transcribed into cDNA using the HiScript Q RT Super-Mix for qPCR Kit (Vazyme Biotech, R323-01, China), following the manufacturer’s instructions. The cDNA was mixed with AceQ qPCR SYBR green master mix (Vazyme Biotech, Q341-02/03) and primers (Supplementary Table 3) for real-time PCR on a StepOnePlus instrument (Applied Biosystems, USA). GAPDH served as an internal control, and relative gene expression levels were calculated using 2^−ΔΔCT^ methods. The primer sequences used for qPCR were listed in Supplementary Table 3.

### Statistical analysis

For the in vitro studies, the data are presented as the mean ± SEM of three independent experiments. The animal study data are presented as the mean ± SEM of the data from four to six mice. For the microglial engulfment and morphology analysis, the data are derived from 10 to 15 microglia per mouse across four biological replicates. Data were analyzed using GraphPad Prism 9.0 software. Unpaired Student’s *t* test applied for comparisons between two groups. For comparisons among multiple groups, one-way analysis of variance (ANOVA) or two-way repeated-measures ANOVA with Tukey’s post hoc test was used. Data are presented as the means ± SEM from at least three independent experiments. Differences were considered significant at *P* < 0.05. Additional details, such as statistical test used, number of samples, and *p* values can be located in the figure legends.

## Supplementary information

Supplementary Information

Supplementary Figure 1

Supplementary Figure 2

Supplementary Figure 3

Supplementary Figure 4

Supplementary Figure 5

Supplementary Figure 6

Supplementary Figure 7

Supplementary Table 1

Supplementary Table 2

Supplementary Table 3

Uncropped western blot images

## Data Availability

The data and materials used in this research are available upon request from the corresponding author.
