# Supplementary material for: Complement receptor 3 (CR3)-dependent microglial synapse elimination drives Parkinson’s disease pathogenesis in systemic inflammation
Source: Cell Death Dis. 2026 Mar 25;17(1):319. doi: 10.1038/s41419-026-08557-9 (PMC13039679; doi:10.1038/s41419-026-08557-9)
Supplement: Supplementary file 1 — Supplementary Information [file 41419_2026_8557_MOESM1_ESM.docx]

**SUPPLEMENTARY MATERIALS**

**Suppl. Fig. 1 The transcriptional levels of Rab5b (A) and Rab7 (B) in the midbrain of LPS-treated mice**. n = 4 mice. Data are presented as mean ± SEM. One-way ANOVA followed by Tukey’s multiple comparisons was used for statistical analysis. **P* < 0.05, ***P* < 0.01, and ****P* < 0.001.

**Suppl. Fig. 2 The transcriptional levels of pro-inflammatory markers (A), purinergic receptors (B), and PNNs components and degradative enzyme (C) in the midbrain of LPS-treated mice**. n = 4 mice. Data are presented as mean ± SEM. One-way ANOVA followed by Tukey’s multiple comparisons was used for statistical analysis. **P* < 0.05, ***P* < 0.01, and ****P* < 0.001.

**Suppl. Fig. 3 The transcription levels of C3 (A) and CR3 (B) in primary microglia after LPS stimulation for 24 h.** n = 3 independent experiments. Data are presented as mean ± SEM. Unpaired t test was used for statistical analysis. **P* < 0.05, ***P* < 0.01.

**Suppl. Fig. 4 The mRNA (A) and protein (B**, **C) levels of CR3 in microglia transfected with siRNA for 48 h.** n = 3 independent experiments. Data are presented as mean ± SEM. Unpaired t test was used for statistical analysis. ***P* < 0.01.

**Suppl. Fig. 5 Effects of CR3 knockdown on the expression of C1q and C3. A**, **B** The transcription levels of C1q (**A**) and C3 (**B**) in primary microglia after LPS stimulation for 24 h. n = 3 independent experiments. **C**, **D** mRNA expression of C1q (**C**) and C3 (**D**) in the midbrain of systemic LPS-induced PD mice. n = 4 mice. Data are presented as mean ± SEM. Two-way ANOVA followed by Tukey’s multiple comparisons was used for statistical analysis. ***P* < 0.01, and ****P* < 0.001.

**Suppl. Fig. 6 Effects of C3/CR3 pathway on microglia induced neuron damage *in vitro*. A**, **B** Quantification of neurons with fragmented nuclei (**A**) and MAP2^+^ cell neurite length (**B**) in VM neurons and microglia-neuron coculture, with or without LPS treatment for 24 h. **C**, **D** Quantification of neurons with fragmented nuclei (**C**) and MAP2^+^ cell neurite length (**D**) in VM neurons coculture with CR3-deficiency microglia. **E**, **F** Quantification of neurons with fragmented nuclei (**E**) and MAP2^+^ cell neurite length (**F**) in microglia-neuron coculture, with or without pegcetacoplan acetate pretreatment. n = 3 independent experiments. Data are presented as mean ± SEM. Two-way ANOVA followed by Tukey’s multiple comparisons was used for statistical analysis. **P* < 0.05, ***P* < 0.01, ****P* < 0.001.

**Suppl. Fig. 7 AAV-mediated depletion of CR3 in microglia.**

**A** Schematic diagram of AAV injection experimental design. **B** Schematic representation of the AAV9 virus construct used to express CR3 or control siRNA in microglia under the Iba1 promoter. **C**, **D** Specificity of AAV infection in the SNc of mice after AAV-NC injection for 4 weeks. Scale bars, 50 µm. n=4 mice. **E**, **F** The overlap of GFP with CR3-negative cells after AAV injection. Scale bars, 50 µm. n=4 mice. **G, H** The expression of CR3 on microglia in the SNc of mice after AAV injection. Scale bars, 50 µm. n=4 mice. Data are mean ± SEM. Unpaired Student’s t test was used for statistical analysis. ***P* < 0.01.

**Suppl. Table. 1 Reagents, commercial kits, and antibodies**

**Suppl. Table. 2 siRNA duplexes used to knockdown specific proteins**

**Suppl. Table. 3 Primers used for RT-PCR**
