# Supplementary figures and images for: Complement receptor 3 (CR3)-dependent microglial synapse elimination drives Parkinson’s disease pathogenesis in systemic inflammation
Source: Cell Death Dis. 2026 Mar 25;17(1):319. doi: 10.1038/s41419-026-08557-9 (PMC13039679; doi:10.1038/s41419-026-08557-9)

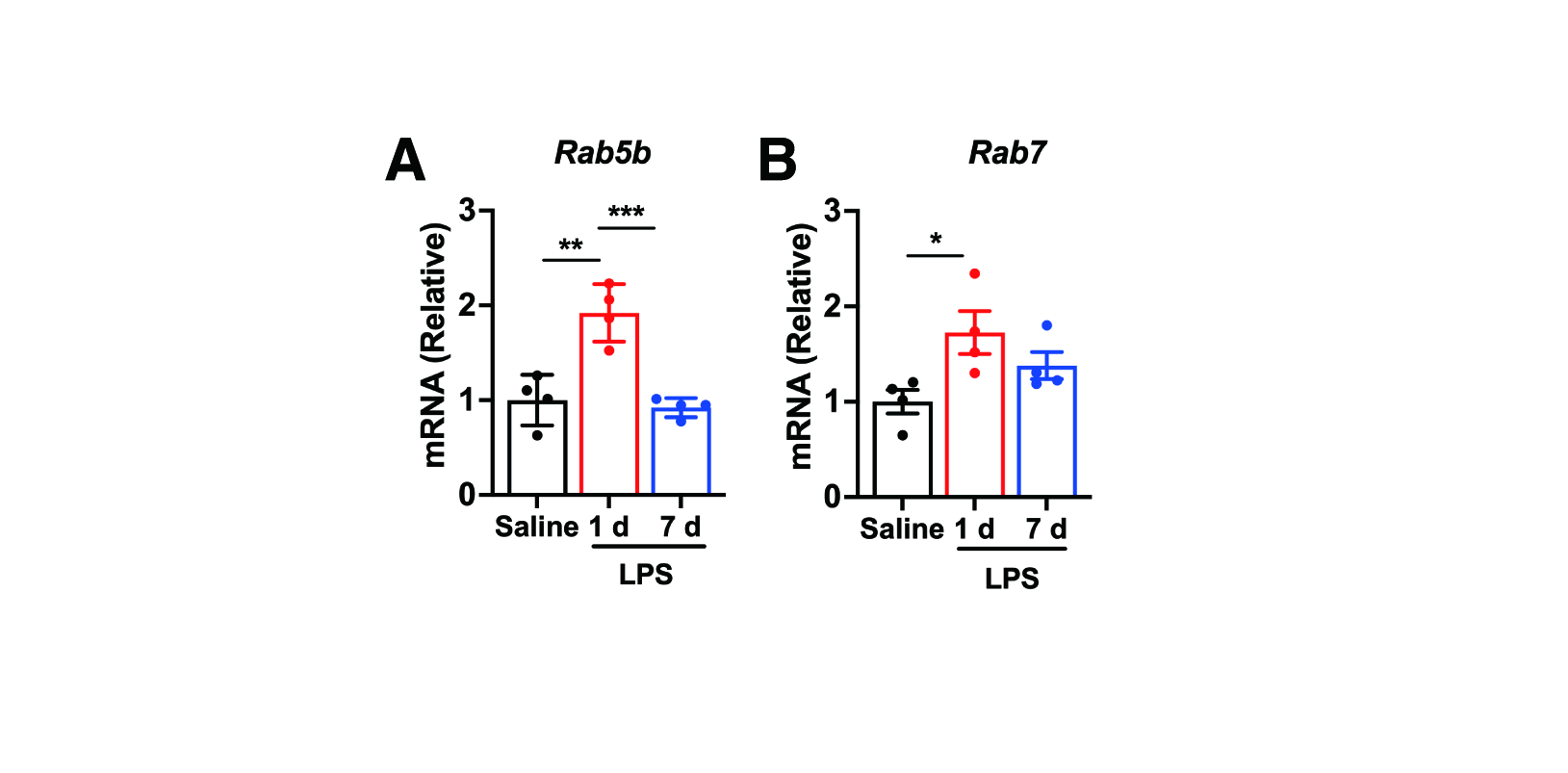

Supplement: Supplementary file 2 — Supplementary Figure 1 [file 41419_2026_8557_MOESM2_ESM.tif]

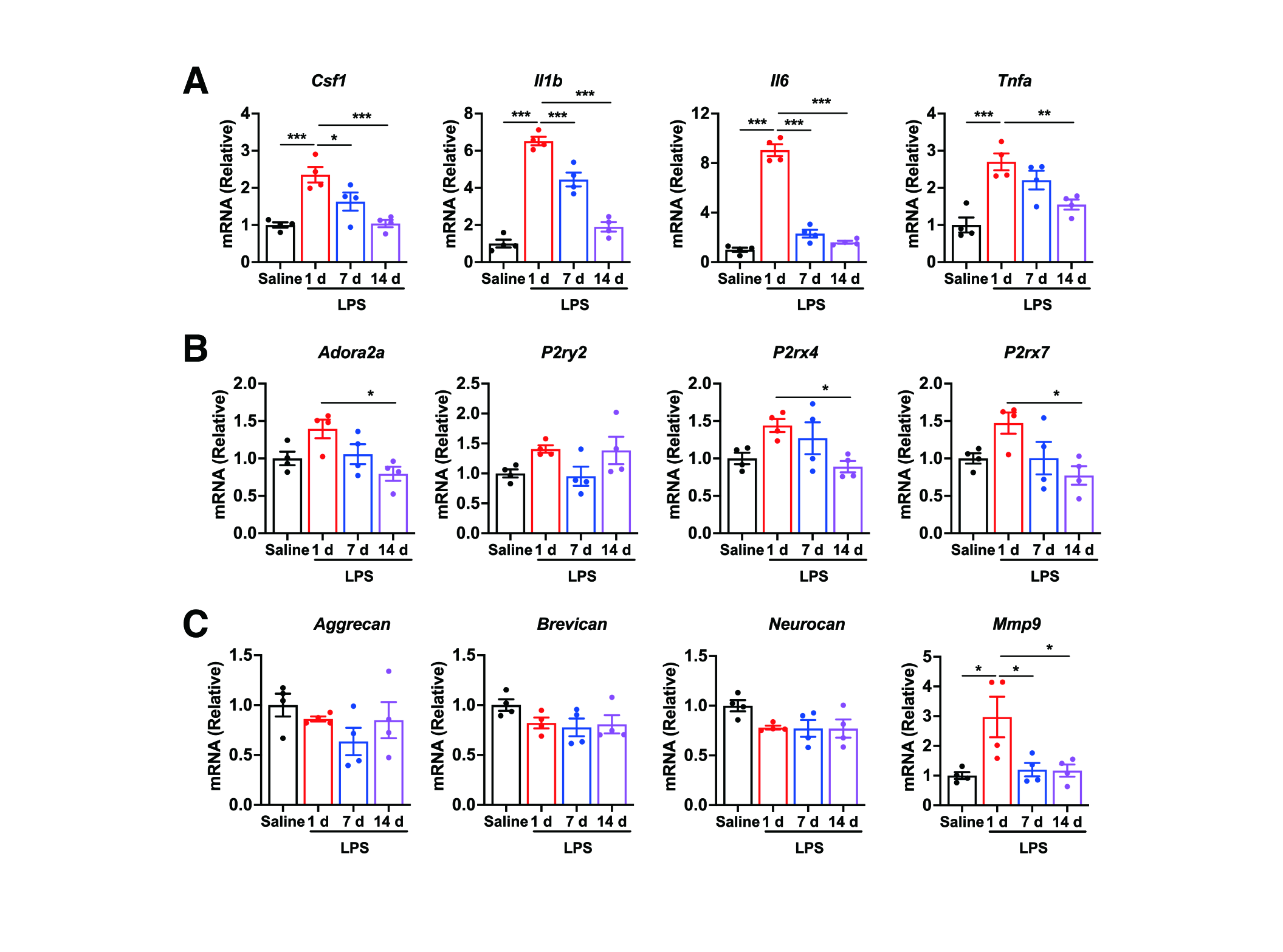

Supplement: Supplementary file 3 — Supplementary Figure 2 [file 41419_2026_8557_MOESM3_ESM.tif]

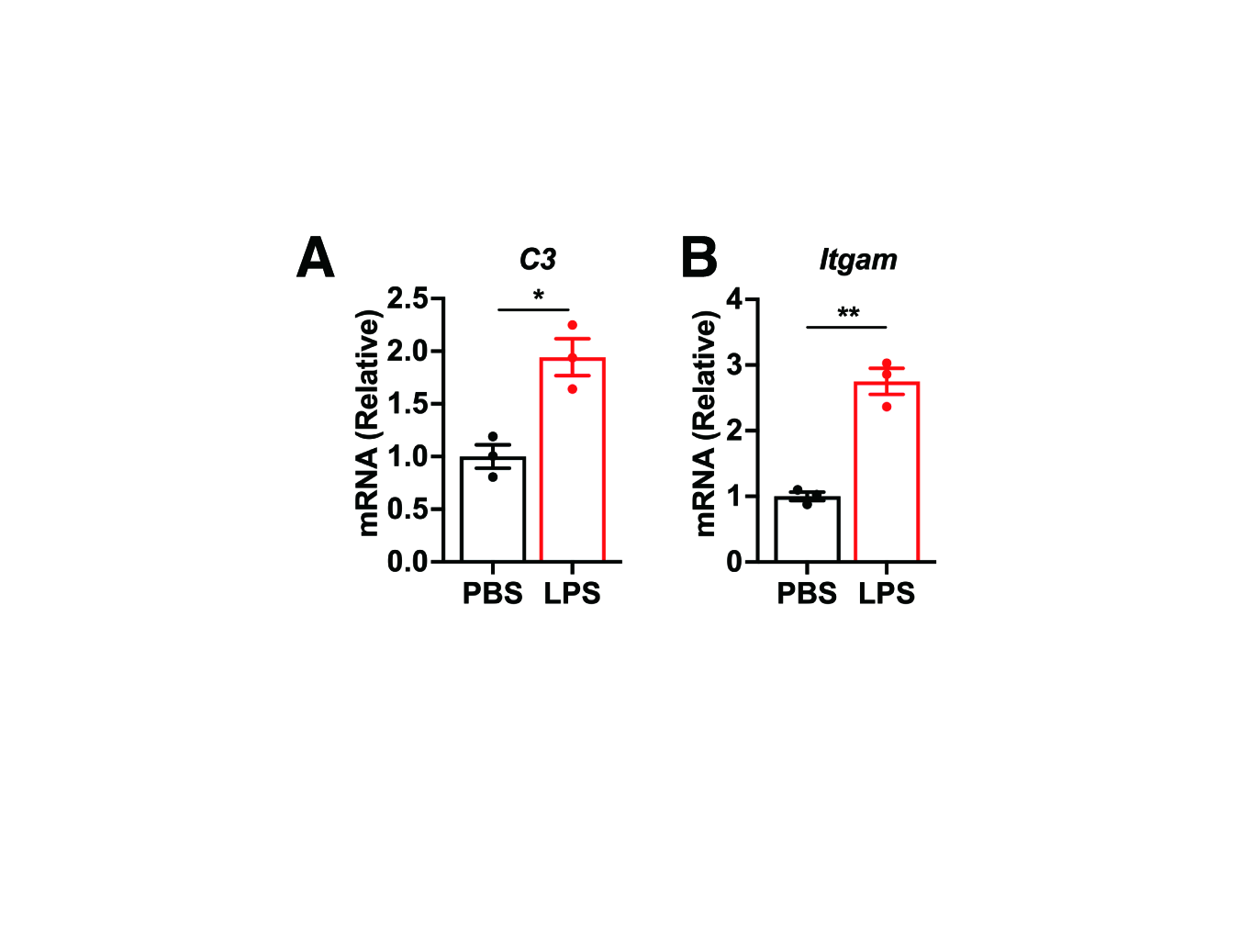

Supplement: Supplementary file 4 — Supplementary Figure 3 [file 41419_2026_8557_MOESM4_ESM.tif]

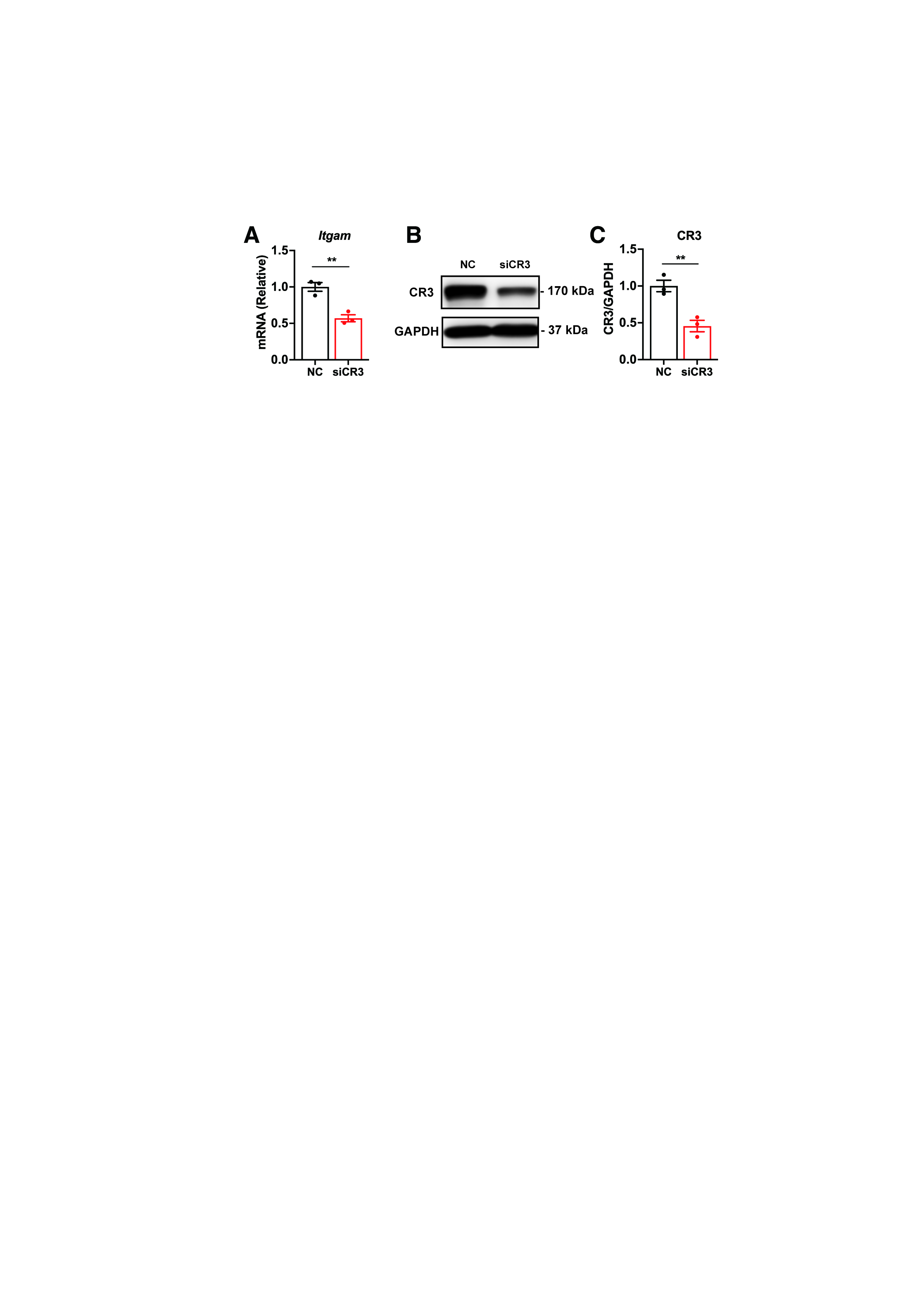

Supplement: Supplementary file 5 — Supplementary Figure 4 [file 41419_2026_8557_MOESM5_ESM.tif]

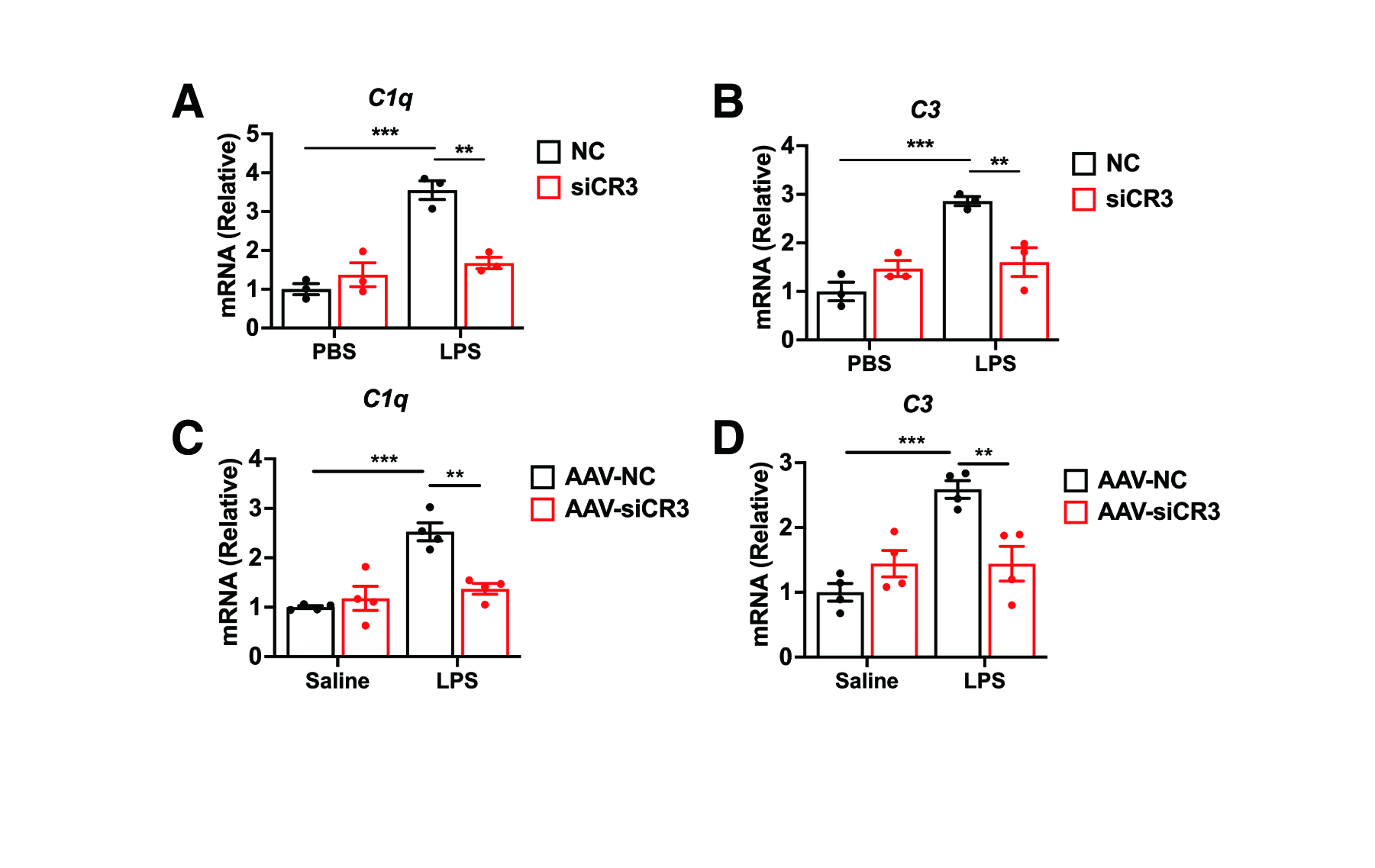

Supplement: Supplementary file 6 — Supplementary Figure 5 [file 41419_2026_8557_MOESM6_ESM.tif]

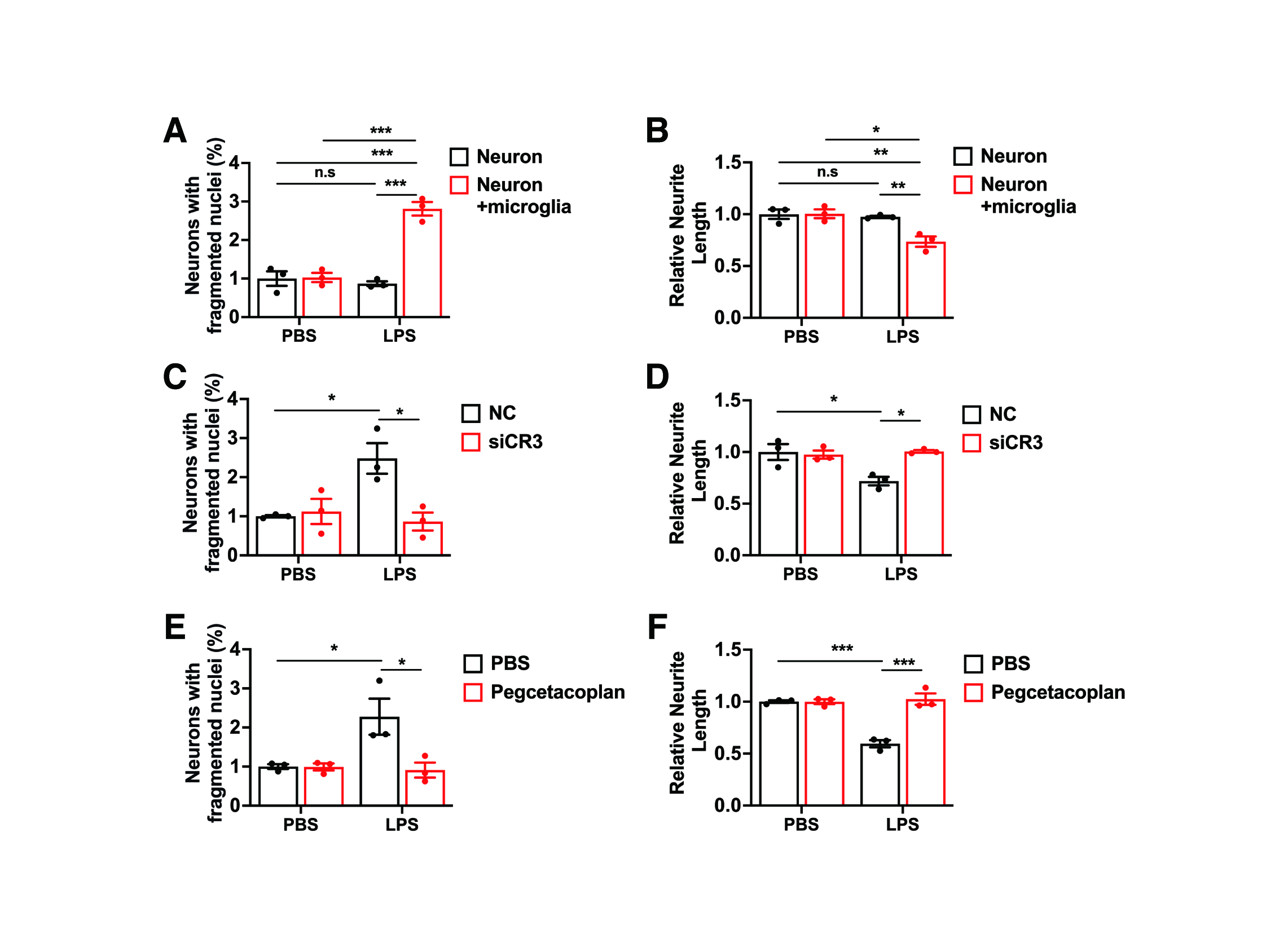

Supplement: Supplementary file 7 — Supplementary Figure 6 [file 41419_2026_8557_MOESM7_ESM.tif]

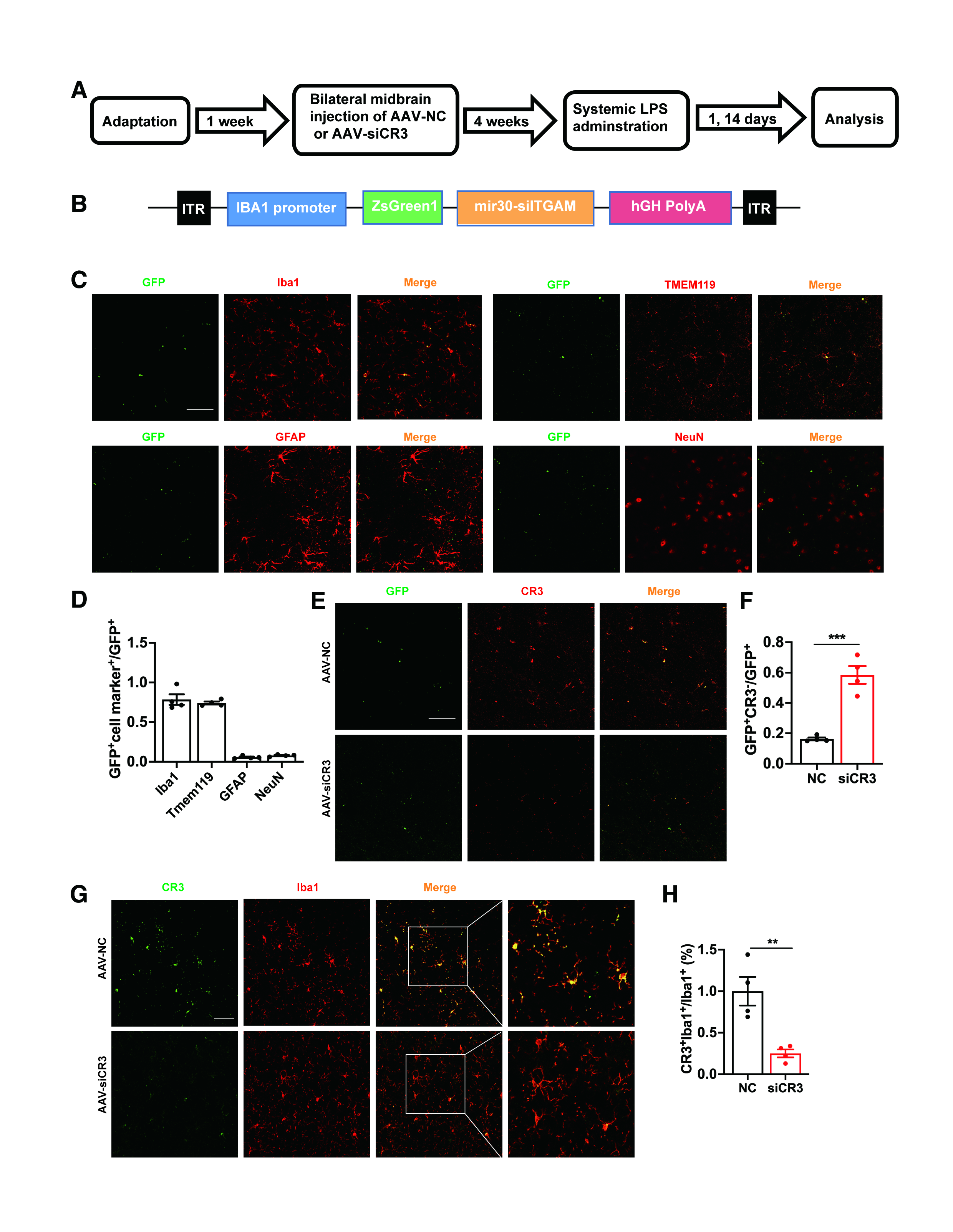

Supplement: Supplementary file 8 — Supplementary Figure 7 [file 41419_2026_8557_MOESM8_ESM.tif]

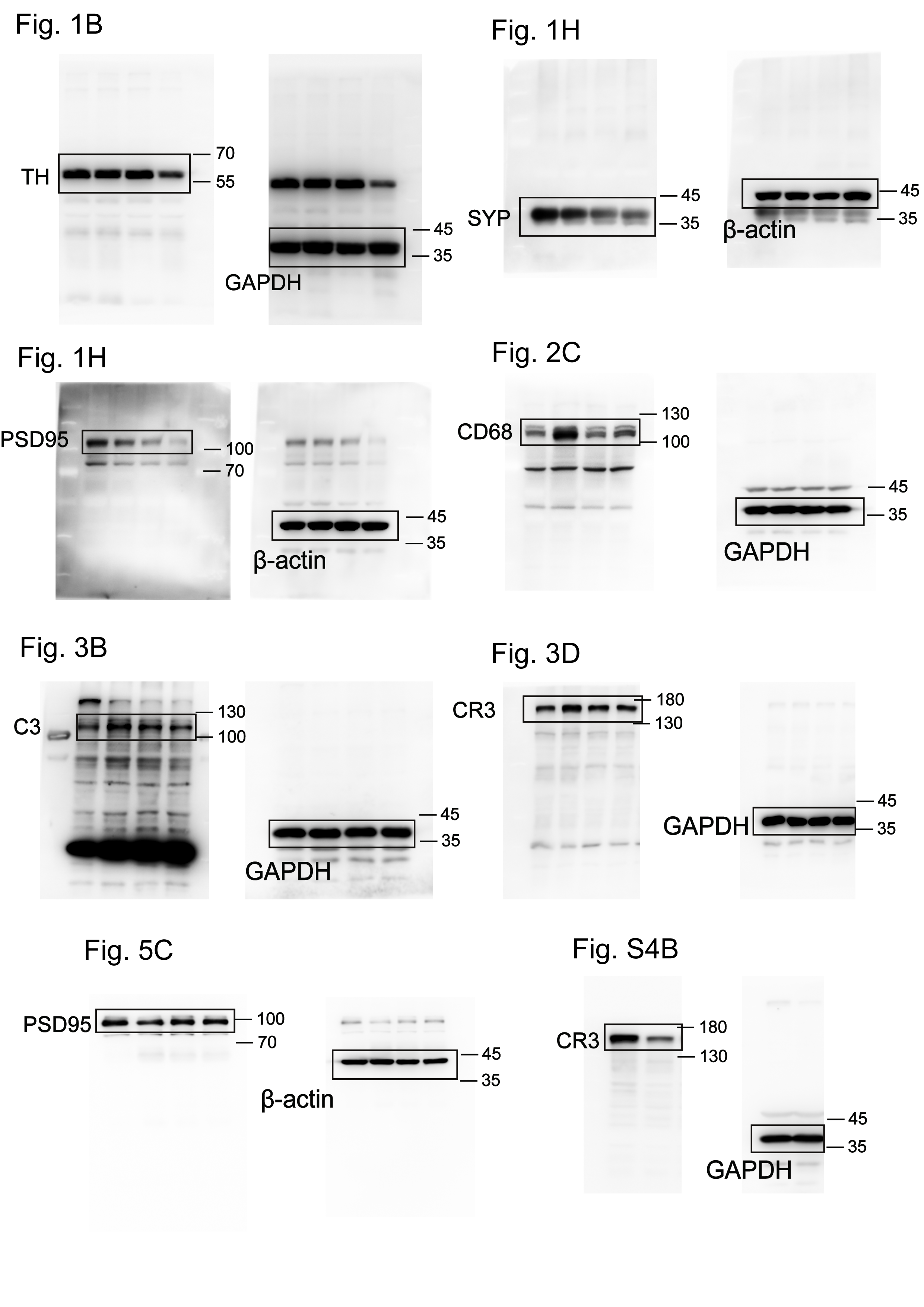

Supplement: Supplementary file 12 — Uncropped western blot images [file 41419_2026_8557_MOESM12_ESM.tif]
