# Supplementary material for: Complement receptor 3 (CR3)-dependent microglial synapse elimination drives Parkinson’s disease pathogenesis in systemic inflammation
Source: Cell Death Dis. 2026 Mar 25;17(1):319. doi: 10.1038/s41419-026-08557-9 (PMC13039679; doi:10.1038/s41419-026-08557-9)
Supplement: Supplementary file 9 — Supplementary Table 1 [file 41419_2026_8557_MOESM9_ESM.docx]

**Table. S1 Reagents, commercial kits, and antibodies**

| **Reagent/Resource** | **Source** | **Identifier or Catalog Number** |
| --- | --- | --- |
| **Antibodies** | | |
| mouse anti-tyrosine hydroxylase (TH) | Santa Cruz Biotechnolog | Cat# sc-25269; RRID:AB_628422 |
| HRP-conjugated goat anti-mouse | Proteintech | Cat# SA00001-1 |
| Rabbit anti- ionized calcium-binding adapter molecule 1 (Iba1) | Wako | Cat# 019-19741; RRID: AB_839504 |
| Mouse anti-Cluster of Differentiation 68 (CD68) | Santa Cruz Biotechnolog | Cat# sc-17832; RRID: AB_627157 |
| Mouse anti-postsynaptic density-95 (PSD-95) | Santa Cruz Biotechnology | Cat# sc-71934; RRID: AB_1128590 |
| Rabbit anti-Synaptophysin (SYP) | Proteintech | Cat# 117785-1-AP, |
| Rabbit anti-C3 | Millipore | Cat# PA5-21349, RRID:AB_11153785 |
| Rabbit anti-ITGAM | Cell Signaling Technology (CST) | Cat# 17800, |
| Alexa Fluor 555 goat anti-mouse | Thermo Fisher Scientific | Cat# A21422;  RRID: AB_141822 |
| Alexa Fluor 488 goat anti-rabbit | Thermo Fisher Scientific | Cat# A11008; RRID: AB_143165 |
| Horseradish peroxidase conjugated anti-mouse | Proteintech | SA00001-1, USA |
| Mouse anti-β-actin | SantaCruz | Cat# sc-8432, RRID:AB_626630 |
| Mouse anti-GAPDH | SantaCruz | Cat# sc-32233,  RRID: AB_627679 |
| **Chemicals, peptides, and recombinant proteins** | | |
| Lipopolysaccharides (LPS) | Sigma-Aldrich | L4516 |
| 0.25% trypsin/EDTA | Solarbio | 9002-07-7 |
| Dulbecco’s modified Eagle medium (DMEM)/F12 medium | Gibco | 12500-062 |
| Fetal bovine serum (FBS) | Gibco | 16000-044 |
| streptomycin-penicillin mixture | NCM Biotech | C125C5 |
| Cell adherent reagent | Applygen | C1010 |
| Poly-L-lysine (PLL) | Sigma-Aldrich | P7405 |
| Neurobasal media | Gibco | 21103-049 |
| B27 supplement | Gibco | 17504044 |
| glutamine | Gibco | 25030081 |
| Lipofectamine RNAiMAX | Invitrogen | 13778-150 |
| Opti-MEM medium | Gibco | 31985-070 |
| Pegcetacoplan acetate | MedChemExpress | HY-P3252A |
| 5% BSA | Yi Fei Xue Bio Technology | TV0815 |
| Goat serum | Boster Biological Technology | 18F29C09 |
| Protease inhibitor cocktail | Invitrogen | A32965 |
| Trizol reagent | Life Ambion | 15596018 |
| AceQ qPCR SYBR green master mix | Vazyme Biotech | Q341-02/03 |
| **Other** | | |
| BCA Protein Assay | KeyGen BioTECH | KPG903 |
| HiScript Q RT Super-Mix for qPCR Kit | Vazyme Biotech | R323-01 |
| DAB staining system | MXB Biotechnologies | DAB-2031 |
| **Software** | | |
| MBF Bioscience Stereo Investigator software | MBF Bioscience | https://www.mbfbioscience.com/ |
| Imaris | Oxford Instruments | https://imairs.oxinst.com/ |
| ImageJ | Schneider et al., 2012 | https://imagej.nih.gov/ij/ |
| Prism 9 | GraphPad | https://www.graphpad.com/scientific-software/prism/ |
