# Supplementary material for: Complement receptor 3 (CR3)-dependent microglial synapse elimination drives Parkinson’s disease pathogenesis in systemic inflammation
Source: Cell Death Dis. 2026 Mar 25;17(1):319. doi: 10.1038/s41419-026-08557-9 (PMC13039679; doi:10.1038/s41419-026-08557-9)
Supplement: Supplementary file 10 — Supplementary Table 2 [file 41419_2026_8557_MOESM10_ESM.docx]

| **Table.S2: siRNA duplexes used to knockdown specific proteins** | | | |  |
| --- | --- | --- | --- | --- |
| Control | sense | UUCUCCGAACGUGUCACGUTT |  | |
|  | antisense | ACGUGACACGUUCGGAGAATT |  | |
| CR3 | sense | CCUGUUUAAUGACUCUGCGUUTT |  | |
|  | antisense | AACGCAGAGUCAUUAAACAGGTT |  | |
|  |  |  | |  |
|  |  |  | |  |
