# Supplementary material for: Complement receptor 3 (CR3)-dependent microglial synapse elimination drives Parkinson’s disease pathogenesis in systemic inflammation
Source: Cell Death Dis. 2026 Mar 25;17(1):319. doi: 10.1038/s41419-026-08557-9 (PMC13039679; doi:10.1038/s41419-026-08557-9)
Supplement: Supplementary file 11 — Supplementary Table 3 [file 41419_2026_8557_MOESM11_ESM.docx]

| **Table.S3: Primers used for RT-PCR** | | | |
| --- | --- | --- | --- |
|  | Forward | Reverse | |
| P2ry12 | 5'-ATGGATATGCCTGGTGTCAACA-3' | 5'-AGCAATGGGAAGAGAACCTGG-3' | |
| Mfge8 | 5'-AGATGCGGGTATCAGGTGTGA-3' | 5'-GGGGCTCAGAACATCCGTG-3' | |
| Cd47 | 5’-TGGTGGGAAACTACACTTGCG-3’ | 5’-CGTGCGGTTTTTCAGCTCTAT-3’ | |
| Sirpa | 5’-CCACGGGGAAGGAACTGAAG-3’ | 5’- ACGTATTCTCCTGCGAAACTGTA-3’ | |
| Mertk | 5’-CAGGGCCTTTACCAGGGAGA-3’ | 5’-TGTGTGCTGGATGTGATCTTC-3’ | |
| Trem2 | 5’-CTGGAACCGTCACCATCACTC-3’ | 5’-GACCCACAGGATGAAACCTGC-3’ | |
| Ifnar1 | 5’-CGTAGCCCCTCAGTGTGT-3’ | 5’-GCCAGCTCCTCCAGTTAGT-3’ | |
| C1q | 5’-AAAGGCAATCCAGGCAATATCA-3’ | 5’-TGGTTCTGGTATGGACTCTCC-3’ | |
| C3 | 5’-CCAGCTCCCCATTAGCTCTG-3’ | 5’-GCACTTGCCTCTTTAGGAAGTC-3’ | |
| Itgam | 5’-ATGGACGCTGATGGCAATACC-3’ | 5’-TCCCCATTCACGTCTCCCA-3’ | |
| Rab7 | 5’-CCTCTGGCTGTGGACAAAAT-3’ | 5’-CTGCTCACAAGAAGCAGTGG-3’ | |
| Rab5a | 5’-CCTCTGGCTGTGGACAAAAT-3’ | 5’-CTGCTCACAAGAAGCAGTGG-3’ | |
| Adora2a | 5’-GCCATCCCATTCGCCATCA-3’ | 5’-GCAATAGCCAAGAGGCTGAAGA-3’ | |
| P2rx4 | 5’-CTGGTGTGCCAACGAGGAATA-3’ | 5’-AGACGGAATATGGGGCAGAAG-3’ | |
| P2rx7 | 5’-GACAAACAAAGTCACCCGGAT-3’ | 5’-CGCTCACCAAAGCAAAGCTAAT-3’ | |
| P2ry2 | 5’-CTGGAACCCTGGAATAGCACC-3’ | 5’-CACACCACGCCATAGGACA-3’ | |
| Aggrecan | 5’-CCTGCTACTTCATCGACCCC-3’ | 5’-AGATGCTGTTGACTCGAACCT-3’ | |
| Brevican | 5’-TGCGCGTCAAGGTAAACGAA-3’ | 5’-GACCCCGGAATCATTGGGC-3’ | |
| Neurocan | 5’-TGCAACCACGGCTAAGCTC-3’ | 5’-GGGGATAAGCAGGCAATGAC-3’ | |
| Mmp9 | 5’-CTGGACAGCCAGACACTAAAG-3’ | 5’-CTCGCGGCAAGTCTTCAGAG-3’ | |
| Il1b | 5’-GCAACTGTTCCTGAACTCAACT-3’ | 5’-ATCTTTTGGGGTCCGTCAACT-3’ | |
| Csf1 | 5’-ATGAGCAGGAGTATTGCCAAGG-3’ | 5’-TCCATTCCCAATCATGTGGCTA-3’ | |
| Il6 | 5’-TAGTCCTTCCTACCCCAATTTCC-3’ | 5’-TTGGTCCTTAGCCACTCCTTC-3’ | |
| Tnfa | 5’-CCCTCACACTCAGATCATCTTCT-3’ | 5’-GCTACGACGTGGGCTACAG-3’ | |
| GAPDH | 5’-AACGACCCCTTCATTGAC-3’ | 5’-TCCACGACATACTCAGCAC-3’ | |
|  |  |  |  |
|  |  |  |  |
|  |  |  |  |
